# Profiling and Targeting of Energy and Redox Metabolism in Grade 2 Bladder Cancer Cells with Different Invasiveness Properties

**DOI:** 10.3390/cells9122669

**Published:** 2020-12-11

**Authors:** Valentina Pasquale, Giacomo Ducci, Gloria Campioni, Adria Ventrici, Chiara Assalini, Stefano Busti, Marco Vanoni, Riccardo Vago, Elena Sacco

**Affiliations:** 1 Department of Biotechnology and Biosciences, University of Milano-Bicocca, Piazza della Scienza 2, 20126 Milan, Italy; valentina.pasquale@unimib.it (V.P.); g.ducci@campus.unimib.it (G.D.); g.campioni@campus.unimib.it (G.C.); a.ventrici@campus.unimib.it (A.V.); stefano.busti1@unimib.it (S.B.); 2 SYSBIO-ISBE-IT-Candidate National Node of Italy for ISBE, Research Infrastructure for Systems Biology Europe, 20126 Milan, Italy; 3 Urological Research Institute, Division of Experimental Oncology, IRCCS San Raffaele Hospital, 20132 Milan, Italy; assalini.chiara@hsr.it; 4 Università Vita-Salute San Raffaele, 20132 Milan, Italy

**Keywords:** bladder cancer, energy and redox metabolism, cellular bioenergetics, mitochondrial function, glycolysis, fatty acids oxidation, oxidative stress, 2D and 3D cultures, Seahorse Extracellular Flux Analyzer, quantitative imaging, Operetta CLS™

## Abstract

Bladder cancer is one of the most prevalent deadly diseases worldwide. Grade 2 tumors represent a good window of therapeutic intervention, whose optimization requires high resolution biomarker identification. Here we characterize energy metabolism and cellular properties associated with spreading and tumor progression of RT112 and 5637, two Grade 2 cancer cell lines derived from human bladder, representative of luminal-like and basal-like tumors, respectively. The two cell lines have similar proliferation rates, but only 5637 cells show efficient lateral migration. In contrast, RT112 cells are more prone to form spheroids. RT112 cells produce more ATP by glycolysis and OXPHOS, present overall higher metabolic plasticity and are less sensitive than 5637 to nutritional perturbation of cell proliferation and migration induced by treatment with 2-deoxyglucose and metformin. On the contrary, spheroid formation is less sensitive to metabolic perturbations in 5637 than RT112 cells. The ability of metformin to reduce, although with different efficiency, cell proliferation, sphere formation and migration in both cell lines, suggests that OXPHOS targeting could be an effective strategy to reduce the invasiveness of Grade 2 bladder cancer cells.

## 1. Introduction

Bladder cancer (BC) is among the most common malignancies worldwide and one of the most expensive cancers to manage [1]. Most BC patients (75–80%) are diagnosed with non-muscle invasive BC (NMIBC). Recurrences are frequent (50–70%), sometimes including progression to invasive tumors (Muscle invasive BC, MIBC), which drastically reduce survival expectations. NMIBC is usually treated by transurethral resection (TUR) to remove the tumor and obtain histological examination material. After resection, patients are introduced to a treatment regimen, which reflects the disease’s nature and potential, based on histological grade and tumor-node-metastasis stage. Therefore, a proper definition of urothelial neoplasms grading is a crucial driver to stratify tumor behavior, including progression and recurrence for optimal patient management and surveillance follow-up [2,3]. In 2004, the World Health Organization (WHO) introduced a classification, confirmed in 2016, including the concept of Low-Grade (LG) and High-Grade (HG) tumors, as well as the papillary urothelial neoplasm of low malignant potential category [4]. One critical issue in this categorization is grade heterogeneity in papillary neoplasms, a mixture of non-invasive HG and LG features that can be observed in around 25% of cases [5]. So far, molecular markers are not part of standard clinical practice, even if molecular changes in HG tumors occur at early stages before developing the corresponding morphological features [6]. In the case of borderline histology, to distinguish LG and HG tumors, other parameters such as urinary cytology, multifocality, size of the lesion, prior history, and recurrence can be considered for grading determination, but they are often not consistent. Mixed lesions represent a challenging topic and no authoritative recommendations on reporting them are available from the WHO 2016 [3].

In this context, it is crucial to identify new markers that can contribute to patients’ stratification to direct them to more effective and less toxic targeted treatments, even to be combined with conventional therapies, to improve prognosis, avoid relapses, or to promote the overcoming of chemoresistance. Reprogramming of energy metabolism has emerged as a hallmark of cancer, and altered metabolic pathways can represent attractive clinical targets exploitable in new therapeutic strategies [7,8]. Cancer cells exhibit profound metabolic rearrangements, which support their enhanced growth, allowing them to proliferate and survive in conditions where normal cells do not [9,10]. Among the most common rearrangements there is the well-known Warburg effect, which consists of enhancing aerobic glycolysis at the expense of mitochondrial respiration, which leads to ferment the glycolysis-derived pyruvate to lactic acid, even under normoxic condition [11]. This metabolic rewiring allows obtaining energy, reducing power and building blocks for biosynthetic processes, faster than through alternative metabolic pathways. This type of metabolism is usually exploited by actively proliferating cells [12,13] and maximally contracted skeletal muscle cells, which must reach short-term high energy goals [14]. Although initially associated with mitochondrial defects, it is clear by now that the Warburg effect also occurs in the presence of functional mitochondria. The activation of specific signaling pathways (i.e., Hypoxia-induced HIF-1α, PI3K-Akt-mTOR, RAS/MEK/MAPK), of specific oncogenes (i.e., RAS [15,16,17], Myc [18]), the inactivation of tumor suppressors (i.e., p53 [19,20]), and/or the selective pressure exerted by the microenvironment [21,22,23,24] all contribute to driving the Warburg effect. The hyperglycolytic phenotype, characterized by increased glucose uptake and glycolysis, is associated with further metabolic changes that include the extensive anaplerotic use of other nutrients, such as glutamine, for the replenishment of the TCA cycle intermediates [25,26,27]. Although these rearrangements can provide selective advantages to cancer cells, they also confer dependence on specific enzymatic activities and specific nutrients and open to novel therapeutic opportunities [28,29,30]. For example, glutamine addicted cancer cells need glutamine to survive because they depend on glutaminolysis for anaplerosis and their biosynthetic needs [27,31]. Many other metabolic targets have been identified in cancers, including urothelial bladder cancers [32,33], and are also evaluated as prognostic markers [34]. Several drugs that affect the altered metabolism are under clinical trial in the perspective of precision medicine [35,36,37]. Deregulations of energy metabolism can also affect reducing equivalents and impact on redox homeostasis by making cancer cells more sensitive to oxidative stress [38,39,40].

In this work we present a characterization of the energy and redox metabolism of two bladder cancer cell lines with a low histological grade of tumor progression (Grade 2), RT112, and 5637. Grade 2 represents a therapeutic window that strongly requires post-surgical resection pharmacological treatments that help to eliminate residual tumor cells and prevent the formation of tumor relapses. Based on gene mutation patterns and genomic changes, the two cell lines are representative of luminal like FGFR3-driven cancer (RT112), and of the basal-like TP53/RB tumor suppressor-driven cancer (5736), often used as models of non-aggressive and aggressive BCs, respectively [41,42]. Here we show that the RT112 cell line is largely less efficient in migration, but forms spheroids more efficiently. Both cell lines produce ATP by both OXPHOS and glycolysis. However, RT112 cells are more energetic, have higher metabolic plasticity and are less sensitive than 5637 to nutritional perturbation of cell proliferation and migration. Although both cell lines, when grown under adhesion are sensitive to targeting of both OXPHOS and glycolysis, although with different efficiency, only targeting of OXPHOS significantly affects spheroid formation of both cell lines.

## 2. Materials and Methods

### 2.1. Materials and Cell Cultures

Grade 2 bladder cancer cell lines RT112 and 5637, originally established from primary urinary bladder carcinoma [43,44], were purchased from American Collection of Cell Cultures (ATCC, Manassas, VA, USA). Cell lines were routinely grown in RPMI-1640 medium (R0883-Merck Life Science, Darmstadt, Germany) supplemented with 10% fetal bovine serum (FBS, Gibco-ThermoFisher, Waltham, MA, USA), 4 mM glutamine, 100 U/mL penicillin and 100 mg/mL streptomycin, at 37 °C in a humidified atmosphere of 5% CO_2_. Cells were passaged using trypsin-ethylenediaminetetraacetic acid (EDTA). Assays on adherent cells were performed in experimental medium: DMEM w/o phenol red (Gibco™-Thermo Fisher Scientific), FBS 10%, 10 mM glucose, 2 mM glutamine, 100 U/mL penicillin and 100 mg/mL streptomycin. Sphere formation was performed in 3D experimental medium DMEM w/o phenol red (Gibco™-Thermo Fisher Scientific), 1% BSA, 10 mM glucose, 2 mM glutamine, 10 µg/mL Insulin (I9278, Merck Life Science), 0.5 µg/mL Hydrocortisone (H0888-1G, Merck Life Science), 20 ng/mL EGF (EGF Human Recombinant, Peprotech, London, UK) 100 ng/mL Cholera Toxin (C8052, Merck Life Science), 100 U/mL penicillin and 100 mg/mL streptomycin).

Anti-β-Actin mouse monoclonal antibody (Catalog number A5441, Sigma-Aldrich, St. Louis, MO, USA), dilution 1:10,000; anti-vimentin mouse monoclonal antibody (Catalog number sc-32322, Santa Cruz Biotechnology, Dallas, TX, USA), dilution 1:2000; Anti-PSrc (Tyr416) rabbit polyclonal antibody (Catalog number 2101, Cell Signaling Technology^®^, Danvers, MA, USA), dilution 1:1000; Anti-Src rabbit polyclonal antibody (Catalog number 2109, Cell Signaling Technology^®^), dilution 1:1000. Unless specified, all reagents were from Merck Life Science. Stocks used: 0.9 M 2-Deoxy-D-glucose (2-DG) in assay medium; 2.5 mM antimycin A in DMSO; 10 mM etomoxir in sterile water; 25 mM carbonyl cyanide *p*-triflouromethoxyphenylhydrazone (FCCP) in DMSO; 1 M H_2_O_2_; 1 M metformin in assay medium; 2.5 mM oligomycin A in DMSO; 2.5 mM rotenone in DMSO; 20 mM UK5099 in DMSO.

### 2.2. Cell Proliferation and Viability Assays

Cell proliferation under H_2_O_2_ treatment was analyzed by growth kinetics: 3.8 × 10^5^ cells were plated in 6-well plates with 2 mL of standard medium and incubated at 37 °C and 5% CO_2_. After 18 h medium was changed and cells were exposed to different perturbed conditions (medium containing different concentrations of H_2_O_2_; 0–50 mM). At different time points the viable cell number was counted using Trypan-Blue exclusion method.

Cell viability under etomoxir treatment was analyzed by an MTT assay: 5 × 10^3^ cells were plated in 96-well plates in 50 µL of standard medium and incubated at 37 °C and 5 % CO_2_. The day after 50 µL of medium supplemented with 2× serial dilutions of etomoxir or vehicle was added to the wells. 72 h after treatment, 20 µL of MTT formazan (20 mg/mL in isopropanol) was added to the culture media. After 1 h of incubation at 37 °C, the medium was gently removed and cells were suspended in 100 µL DMSO and then absorbance at 570 nm was recorded by using a Victor Multilabel Plate Reader (Perkin–Elmer, Waltham, MA, USA). The viability of cells treated with increasing concentrations of drugs was tested relative to the viability of the same cells treated with vehicle.

Cell proliferation in response to metabolic targeting was analyzed by growth kinetics: 2.1 × 10^4^ cells were plated in Cell Imaging 24-well Plates in 500 µL of experimental medium at 37 °C and 5% CO_2_. After 24 h medium was changed, and cells were exposed to experimental medium containing dose-response concentrations of selected drugs (2DG 0-0.5-5-15 mM, metformin 0-1-5-10-20 mM). Cell growth was monitored through imaging acquisitions every 24 h using Operetta CLS™ high-content analysis system in brightfield using 10× magnification. After 72 h cells were stained with Hoechst 33342 (working concentration 1 ug/mL incubated for 15 min at 37 °C and 5% CO_2_) and imaging acquisitions were made using Operetta CLS™ with 10× magnification in brightfield and widefield fluorescence microscopy. Total cell count (nuclei positive for Hoechst 33342) was obtained using the Harmony software.

### 2.3. Wound Healing Assay

Cell migratory capacity was evaluated with wound healing assay. 10^5^ cells were plated in Cell Imaging 24-well Plates in 500 µL of experimental medium at 37 °C and 5% CO_2_. As an adherent confluent monolayer formed, cells were stained using CellTracker™ Red CMTPX Dye 5 µM (C34552, stock 10 mM in DMSO, Invitrogen™-Thermo Fisher Scientific), according to the supplier’s instructions, to track cell movements. Cell starvation lasting 8 h, with FBS-free experimental medium, was performed to stop cell proliferation. At the end of starvation protocol, pre-wound images of the entire well were acquired using Operetta CLS™ using 10× magnification in brightfield and widefield fluorescence microscopy to detect CellTracker. Next, a wound was made by scratching the monolayer cells with a sterile 10 µL pipette tip, and, after washing, the medium was changed to 0.1% FBS experimental medium containing dose-response concentrations of selected drugs (2DG 0-0.5-5-15 mM, metformin 0-1-5-10 mM). Wound coverage was monitored with automatized time-lapse imaging acquisition using Operetta CLS™, acquiring every entire well of the 24-well plate, both in brightfield and widefield fluorescence microscopy, using 10× magnification, every 35 min (time of acquisition of the entire plate) for 40 h with monitored temperature (37 °C) and atmosphere (5% CO_2_). Evaluation of drug-dependent wound coverage was obtained using the Harmony software.

### 2.4. Sphere Formation Assays

Sphere formation was evaluated using different cell growth supports: 6-well plates not treated for cell adhesion, 6-well cell-repellent plates and Cell Imaging 24-well Plates coated with Poly(2-hydroxyethyl methacrylate) (Poly-HEMA, Merck Life Science). 4 × 10^5^ cells in 6-well and 1.5 × 10^5^ cells in 24-well were seeded in 3D experimental medium and sphere formation was monitored every 24 h with phase contrast microscopy and with Operetta CLS™, respectively, until the endpoint (72 h). We monitored sphere formation with imaging analysis. 4 × 10^4^ cells were seeded on CellCarrier-96 ULA Ultra Microplates 96-well (PerkinElmer) pre-stained with CellTracker™ Red CMTPX Dye 5 µM (Invitrogen™-Thermo Fisher Scientific). Assay was performed in 200 µL 3D experimental medium containing dose-response concentrations of selected drugs (2DG 0-0.5-5-15 mM, metformin 0-1-5-10-20 mM). Sphere morphology was monitored through imaging acquisitions of every complete well every 24 h, for 72 h, using Operetta CLS™, both in brightfield and widefield fluorescence microscopy with 10× magnification. We analyzed sphere formation using the Harmony software.

### 2.5. Metabolic Profiling by Seahorse Assays

Bioenergetic parameters of RT112 and 5637 cells under standard and nutritionally perturbed growth conditions were measured with the Seahorse Extracellular Flux XF24 and XFe96 analyzers (Agilent, Santa Clara, CA, USA; https://www.agilent.com/en/products/cell-analysis/how-seahorse-xf-analyzers-work). *XF Glycolysis stress test, Mitochondrial stress test*, and *Palmitate-BSA Fatty Acid Oxidation assay kit* protocols (Agilent) were performed using the XF24 analyzer, while *ATP rate assay* and *Mitochondrial Fuel Flex test kit* protocols (Agilent) were performed with the XFe96 analyzer, according to the manufacturer’s instructions. Briefly cells were seeded in Seahorse XF plates at a density of 5 × 10^4^ (XF24) or 4 × 10^4^ (XFe96) cells per well and cultured for 24 h. The next day medium was replaced with low buffered XF assay medium (103575-100 Agilent), supplemented with 10 mM glucose, unless otherwise specified, and 2 mM glutamine and cell cultures were allowed to equilibrate for 1 h at 37 °C in a no-CO_2_ incubator. Seahorse XF analysis was performed at 37 °C simultaneously measuring Oxygen Consumption Rate (OCR = pmole O_2_/min) and ExtraCellular Acidification Rate (ECAR = mpH/min). The XFe96 analyzer allows also measuring the Proton Efflux Rate (PER = pmolesH^+^/min). At the end of the analysis performed in XF24 plates, the medium was removed, cells were gently washed with PBS, suspended in JS lysis buffer and scraped after two freezing and thawing cycles. The protein content of cell lysates was measured by Bradford assay and used to normalize respiratory and glycolytic parameters. Subsequently these parameters were normalized on cell number, taking into consideration the protein content per cell. For the assays in XFe96 format, at the end of the Seahorse measurements, Hoechst 33342 was added to each well at the final working concentration of 1 ug/mL and after 15 min incubation nuclei/well were imaged and counted by Operetta CLS™ software Harmony, and directly used to normalize the Seahorse parameters per cell number. Samples were analyzed with at least 10 technical replicates. Data derive from two independent experiments.

#### 2.5.1. XF Cell Mitochondrial Stress Test

This assay was performed to determine the mitochondrial bioenergetics. Oxygen Consumption Rate was analyzed under basal condition and after the treatment with different drugs including the ATP synthase inhibitor oligomycin A (optimal dose chosen after dose-response optimization assay: 0.125–2 µM oligomycin A), an ETC accelerator ionophore (optimal dose chosen after dose-response optimization assay: 0.25–4 µM FCCP), and an ETC inhibitors mixture (1 µM rotenone and 1 µM antimycin A). The response to the minimal dose of oligomycin A and FCCP, generating the maximal effect, accounts for non-phosphorylating mitochondrial respiration and maximal FCCP-uncoupled respiration, respectively. The response to the rotenone and antimycin A mixture accounts for non-mitochondrial oxygen consumption. Respiratory parameters were elaborated using the following formulas:

Basal Mitochondrial Respiration (Basal-MR) = OCR_basal_ − OCR_rot/ant_

Non-Phosphorylating Mitochondrial Respiration (oligo-NPMR) = OCR_oligo_ − OCR_rot/ant_

FCCP-uncoupled Mitochondrial Respiration (FCCP-MR) = OCR_FCCP_ − OCR_rot/ant_

Sare respiratory capacity = OCR_FCCP_ − OCR_rot/ant_/OCR_basal_ − OCR_rot/ant_

Coupling efficiency = 1 − (OCR_oligo_ − OCR_rot/ant_/OCR_basal_ − OCR_rot/ant_)

#### 2.5.2. XF Glycolysis Stress Test

This assay was performed to determine the glycolytic bioenergetics. Extracellular acidification was analyzed in glucose-free-medium before and after the sequential injections of 10 mM glucose, oligomycin A (optimal dose chosen after dose-response optimization assay: 0.125–2 µM oligomycin A), and 50 mM 2-Deoxy-D-glucose (2-DG), a glycolysis inhibitor. The response to the minimum dose generating the maximal effect of oligomycin A accounts for glycolytic capacity. The response to 2-DG accounts for non-glycolytic extracellular acidification. Data were elaborated using the following formulas:

Basal glycolysis = ECAR_glc_ − ECAR_2-DG_

Glycolytic capacity = ECAR_oligo_ − ECAR_2-DG_

Glycolytic reserve = ECAR_oligo_ − ECAR_glc_

#### 2.5.3. XF ATP Rate Assay

This assay measures OXPHOS and glycolysis’s contribution to ATP production. This assay was performed under standard and 2 h glucose deprivation. OCR and acidification were measured before and after sequential injection of 1.5 µM oligomycin A and 0.5 µM rotenone/antimycin A. Data were assessed with XF Wave Software (Seahorse Bioscience, Agilent).

#### 2.5.4. XF Palmitate-BSA FAO Assay

Fatty Acid Oxidation (FAO) of RT112 and 5637 cells were analyzed using the XF Palmitate-BSA FAO Substrate, namely 1 mM palmitate, a long chain fatty acid, conjugated to 0.17 mM BSA (6:1 palmitate:BSA ratio). For the FAO assay 3.5 × 10^5^ cells were seeded in 100 µL of the standard medium onto Seahorse XF24-well plates and incubated at 37 °C and 5% CO_2_. After 24 h, medium was replaced with substrate-limited medium (DMEM medium supplemented with 0.5 mM glucose, 1 mM glutamine, 0.5 mM carnitine and 1% FBS, 5 mM Hepes pH 7.4) and cells were incubated at 37 °C, and 5% CO_2_. After 24 h (45 min before XF analysis), the medium was replaced with FAO-assay medium (KHB supplemented with 5 mM glucose, 0.5 mM glutamine, 0.5 mM carnitine and 5 mM Hepes pH 7.4) and cell cultures were allowed to equilibrate for 1 h at 37 °C in a no-CO_2_ incubator. After 30 min (15 min before XF analysis) 40 µM etomoxir or vehicle was added to appropriate wells on the microplate, and just before initiating XF analysis 220 µM palmitate-BSA or BSA was added to appropriate wells on the microplate. In summary we tested four different conditions for each XF FAO assay:

(1) BSA-Eto (BSA control without etomoxir) accounting for total respiration, including that one deriving from endogenous fatty acids oxidation;

(2) BSA+Eto (BSA control with etomoxir) accounting for respiration not depending on endogenous fatty acids oxidation;

(3) Palm:BSA-Eto (palmitate-BSA without etomoxir) accounting for total respiration, including that one deriving from exogenous fatty acids oxidation;

(4) Palm:BSA+Eto (palmitate-BSA with etomoxir) accounting for respiration not depending on exogenous fatty acids oxidation.

#### 2.5.5. XF Mitochondrial Fuel Flex Test

This assay was performed to measure the dependency, capacity and flexibility of cells to oxidize three critical mitochondrial fuels—glucose, glutamine and fatty acids. The assay was performed in an XF assay medium containing 10 mM glucose, 2 mM glutamine and 1 mM Na-pyruvate. Cells were exposed to BPTES (3 μM), etomoxir (4 μM) or UK5099 (2 μM) in succession and OCR was measured before and after injection of each compound. Data were assessed with XF Wave Software (Seahorse Bioscience, Agilent).

### 2.6. Flow Cytometry

For FACS analysis 1 × 10^6^ cells were plated in 6-well plates with 2 mL of experimental medium and incubated overnight at 37 °C and 5% CO_2_. Cells were then stained with different dyes, as described below, before or after treatment with trypsin-EDTA following dye and antibody protocols and the supplier’s recommendations, suspended in PBS supplemented with 0.2% BSA and acquired by CytoFlex S (Beckman Coulter, Brea, CA, USA). Data were elaborated using CytExpert 2.0 software (Beckman Coulter).

To evaluate mitochondrial membrane potential and mass cells were stained with 100 nM MitoTracker™ Red CMXRos (Invitrogen™ Thermo Fisher Scientific, 1 mM stock in DMSO) and 25 nM MitoTracker™ Green FM (Invitrogen™ Thermo Fisher Scientific, 1 mM stock in DMSO), respectively, in FBS-free medium for 20 min at 37 °C and 5% CO_2_.

To evaluate mitochondrial and intracellular ROS, cells were stained with 5 µM MitoSOX™ Red Mitochondrial Superoxide Indicator (MitoSOX, Invitrogen™ Thermo Fisher Scientific, 5 mM stock in DMSO), and 10 mM 2′,7′-dichlorodihydrofluoresceine diacetate (H2DCFDA Merck Life Science, 5 mM stock in ethanol), respectively, in FBS-free medium for 30 min at 37 °C and 5% CO_2_. According to fluorescence panel necessity, dead cells were excluded from analysis using viability dyes 7-aminoactinomycin D (Thermo Fisher Scientific) or LIVE/DEAD™ Fixable Green Dead Cell Stain (Thermo Fisher Scientific). Cell staining was performed according to supplier’s instruction.

To evaluate stemness, cells were stained as single cell suspension with conjugated antibody CD44-APC (cod. 17-0441-82 eBioscience™, San Diego, CA, USA), CD133-PE (cod. 12-1338-42 eBioscience™), and Aldefluor, Stem Cell Identification Kit (STEMCELL Technologies™, Vancouver, BC, Canada) according to supplier’s protocol. Dead cells were excluded from analysis using viability dyes 7-aminoactinomycin D.

Data were expressed as Median Fluorescence Intensity (MFI) of labeled living cells corrected for MFI of unlabeled living cells’ autofluorescence.

### 2.7. Imaging

Analysis of mitochondrial machinery and redox status was performed by high-resolution imaging.

#### 2.7.1. High-Resolution Imaging for Quantitative Analysis

Cells (6.5 × 10^4^) were seeded per well on Cell Imaging 24-well Plates, incubated overnight at 37 °C and 5% CO_2_. The day after cells were stained with selected dye, then gently washed with phenol red-free medium and promptly analyzed in the experimental medium using Operetta CLS™, with confocal imaging set-up and 63× magnification. To evaluate mitochondrial membrane potential and mass, cells were stained with 100 nM MitoTracker™ Red CMXRos and 25 nM MitoTracker™ Green FM, respectively, for 20 min at 37 °C and 5% CO_2_.

To evaluate mitochondrial ROS, intracellular ROS and lipid oxidation status, cells were stained with 3 µM MitoSOX (5 mM stock in DMSO), 10 µM H2DCFDA (20 mM stock in DMSO) and 10 µmM BODIPY™ 581/591 C11 (Lipid Peroxidation Sensor, Invitrogen™ Thermo Fisher Scientific, 20 mM stock in DMSO), respectively, for 25, 10, and 30 min at 37 °C and 5% CO_2_. Following imaging acquisition of selected dye, cells were stained with Hoechst 33342 (1 µg/mL incubated for 15 min at 37 °C and 5% CO_2_) and further imaging acquisitions were made using Operetta CLS™ with 63× magnification with the confocal set-up. Dye quantitative analysis and total cell count (nuclei positive for Hoechst 33342) were obtained using Harmony software (as schematized in Supplementary Figure S1D–F).

To analyze mitochondrial mass, we plated 1.2 × 10^4^ cells in CellCarrier-96 Ultra Microplates 96-well (PerkinElmer) and incubated at 37 °C and 5% CO_2_ overnight. The day after, cells were transiently transfected using Lipofectamine reagent (Invitrogen, Carlsbad, CA, USA), according to the manufacturer’s instruction, with 1.0 µg of pEYFP-mito vector expressing the mitochondrially targeted yellow fluorescent protein MitoYFP (Clontech, Mountain View, CA, USA). After 48 h, cells were stained with Hoechst 33342 (working concentration 1 ug/mL incubated for 15 min at 37 °C and 5% CO_2_) and imaging acquisitions were made using Operetta CLS™ with 63× magnification with the confocal set-up. Quantitative analysis and total cell count (nuclei positive for Hoechst 33342) were obtained using the Harmony software.

For cell area measurements, 6.5 × 10^4^ cells were seeded per well on Cell Imaging 24-well Plates, incubated overnight at 37 °C and 5% CO_2_. The day after cells were stained using Vybrant™ DiI Cell-Labeling Solution 5 µM (V22885, stock solution 1 mM, Invitrogen™-Thermo Fisher Scientific) according to supplier’s instruction, then analyzed in the experimental medium using Operetta CLS™ with confocal imaging set-up and 63× magnification. Cell area was obtained using the Harmony software.

#### 2.7.2. High-Resolution Imaging with Manually Operated Confocal Microscopy

To analyze SOX2 expression in cells grown as monolayers or spheroids, cells were seeded in different 6-well plates: 1 × 10^5^ cells for RT112 cells and 1.2 × 10^5^ cells for 5637 cells in tissue culture-treated plates in 2 mL 2D experimental medium and 4 × 10^5^ cells for both RT112 and 5637 cells in not-treated and cell repellent plates in 2 mL 3D experimental medium and incubated at 37 °C and 5% CO_2_. Sphere formation was monitored every 24 h with phase contrast microscopy until endpoint (72 h). Adherent cells were harvested by trypsinization and spheres were dissociated. Cells were seeded at subconfluent concentration on 13 mm microscopy slides and incubated at 37 °C, 5% CO_2_ for 24 h. Cells were fixed in two passages with progressive higher concentration of paraformaldehyde at room temperature. After washing with PBS, blocking solution (PBS 1×, 10% normal goat serum (NGS), 0.2% Triton X) was added, incubation lasted for 1 h and 30 min at room temperature. Incubation with primary antibody 1:100 in PBS 1×, NGS 10% was performed overnight at 4 °C (anti-h/mSOX2 MAB2018 Clone 245610 R&D Systems, Minneapolis, MN, USA). After washing with PBS, each slide was incubated with secondary antibody 1:1000 (anti-mouse IgG2a conjugated PE) in the same buffer for 45 min at room temperature in an obscured chamber. Cells were stained with Hoechst 33342 (0.5 ug/mL incubated for 15 min at RT and 5% CO_2_) and image acquisitions were made using an A1R confocal microscope (Nikon, Tokio, Japan) with 40× magnification.

For high resolution imaging of mitochondrial machinery, 2 × 10^4^ cells were plated in 10 compartment CellView culture slide with glass bottom (GreinerBioOne, Kremsmünster, Austria) and incubated overnight at 37 °C and 5% CO_2_. Afterward, cells were stained as previously described with MitoTracker™ Green FM and MitoTracker™ Red CMXRos and analyzed by live imaging confocal microscopy with a Nikon A1R confocal microscope for 48 h with 63× magnification.

For analysis of mitochondrial network morphology, 2.5 × 10^5^ cells were plated in Cellview cell culture dishes with glass bottom (Greiner BioOne) and incubated at 37 °C and 5% CO_2_ overnight. The day after, cells were transiently transfected using Lipofectamine reagent (Invitrogen), according to the manufacturer’s instruction, with 1 µg of pEYFP-Mito vector. Forty-eight h after transfection cells were stained with Hoechst 33342 (1 μg/mL incubated for 15 min at 37 °C and 5% CO_2_) and analyzed by live imaging with a Nikon A1R confocal microscope with 100× magnification.

### 2.8. RNA Extraction and qRT-PCR

Total RNA from bladder cancer cells was extracted using TRIzol LS Reagent (Invitrogen) according to the manufacturer’s recommended protocols. The total RNA quantity was assessed using a Nanodrop spectrophotometer (ND-1000, Nanodrop, Labtech International, Uckfield, UK). Each RNA sample was then retrotranscribed with High Capacity cDNA Reverse Transcription Kit (Applied Biosystems, Foster City, CA, USA). A calibration curve was performed for each gene of interest to test primers and to determine the correct concentration of cDNA to be used for quantitative real time PCR. Reactions were run on an ABI 7000 Real Time PCR system (Applied Biosystems). Primers were purchased from Eurofins (Luxembourg). ATP5A1, ATP5B1, ALDH3A1, GAPDH, LDHA, PKM1 and PKM2 expression levels (indicated as “fold change”) were analyzed in triplicate, normalized to HPRT1 and calculated according to the CT method. Primers used: ATP5A1F 5′-CATTGGTGATGGTATTGCGC-3′; ATP5A1R 5′-TCCCAAACA CGACAACTCC-3′; ATP5B1F 5′-CCGTGAGGGCAATGATTTATAC-3′; ATP5B1R 5′-GTCAAAC CAGTCAGAGCTACC-3′; ALDH3A1-F 5′-GCAGACCTGCACAAGAATGA-3′ALDH3A1-R 5′-TGTAGAGCTCGTCCTGCTGA-3′; GAPDH-F 5′-GGACTCATGACCACAGTCCA-3′, GAPDH-R 5′- CCAGTAGAGGCAGGGATGAT-3′; LDHA-F5′-AGCCCGATTCCGTTACCT-3′, LDHA-R 5′-CA CCAGCAACATTCATTCCA-3′; PKM1-F5′-ACCGCAAGCTGTTTGAAGAA-3′, PKM1-R 5′-TCCA TGAGGTCTGTGGAGTG-3′; PKM2-F 5′-ATCGTCCTCACCAAGTCTGG-3′, PKM2-F 5′-GAAG ATGCCACGGTACAGGT-3′; HPRT1-F 5′-TGCAGACTTTGCTTTCCTTG-3′, HPRT1-R 5′- CTGGCT TATATCCAACACTTCG-3′.

### 2.9. Protein Content, Western Blotting

To determine the cell size of RT112 and 5637 cells, we measured the protein content by Bradford assay (Bio-Rad, Hercules, CA, USA) on cell lysates obtained from 1 × 10^6^ cells harvested by trypsinization, gently washed in PBS, suspended in lysis buffer (50 mM Tris-HCl pH 7.4, 5 mM EDTA, 1 mM EGTA, 10 mM 2-mercaptoethanol), and subjected to three freeze and thaw cycles. For western blotting analysis, 1 × 10^6^ cells were seeded in p100 plates in complete medium and incubated overnight for the attachment. After 24h cells were washed with phosphate buffer saline without calcium and magnesium (PBS), scraped and lysed in lysis buffer supplemented with protease and phosphatase inhibitors. Proteins quantification was performed using BIO-RAD BCA Protein Assay Kit. Cellular lysates (10–30 μg proteins) were resuspended in Sample Buffer with β-mercaptoethanol (312.5 mM Tris-HCl pH 6.8, 10% SDS, 50% glycerol, 25% β-mercaptoethanol and 0.01% bromophenol blue) and analyzed by SDS-PAGE. After electrophoresis, proteins were transferred to nitrocellulose membrane by electroblotting. The membrane was incubated 1 h or overnight with 5% nonfat milk in Tris Buffered Saline supplemented with Tween 20 (TBS-T): 10 mM Tris, pH 8.0, 150 mM NaCl, 0.1% Tween 20. Membranes were probed with specific antibodies for 1 h or overnight. Blots were washed with TBS-T for three times and incubated with ECL (Amersham ECL Prime Western Blotting Detection Reagent, GE Healthcare UK, Little Chalfont, UK) according to the manufacturer’s protocols. Bands were analyzed with the ImageJ software.

### 2.10. Statistical Analysis

Unless specified otherwise, all experiments were carried out at least in triplicate. The number of technical replicates within each experiment is reported in the Results section. Results were expressed as means ± standard deviation and variables were compared using unpaired Student’s *t*-test or general linear model according to their distribution. A *p*-value < 0.05 was considered statistically significant. All statistical analyses were performed using GraphPad version 6 (GraphPad Software, Inc., San Diego, CA, USA) software.

## 3. Results

### 3.1. Morpho/Functional Features of RT112 and 5637 Grade 2 Bladder Cancer Cells

**Figure 1:**
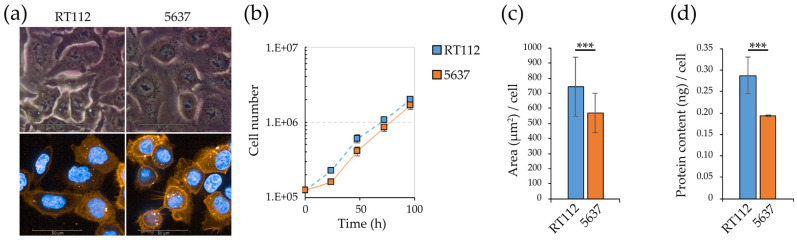

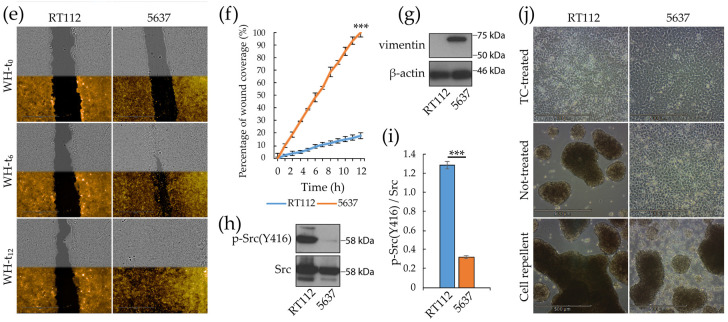
Morpho/functional readouts of RT112 and 5637 cells: proliferation rate, migration and spheroids formation capacity. (**a**) RT112 and 5637 cell morphology by phase contrast and confocal microscopy. For confocal microscopy cell membranes and nuclei were stained with fluorescent Vybrant™ DiI Cell-Labeling Solution (orange) and Hoechst 33342 (blue) dye respectively. (**b**) RT112 and 5637 cell growth curves in standard medium. Viable cells were counted by the Trypan-Blue exclusion method. (**c**) RT112 and 5637 average cell surface area measured as Vybrant™ fluorescent area using Harmony software. (**d**) RT112 and 5637 average protein content per cell measured by Bradford assay. (**e**) Representative wound healing assay images acquired by brightfield and fluorescence microscopy with Operetta CLS™ at 0 h, 6 h and 12 h after scratch (orange: CellTracker™ Red CMTPX Dye). (**f**) Percentage of wound coverage in wound healing assay through time (0–12 h), measured using Harmony software. (**g**,**h**) Western blot analysis for vimentin (**g**) and for Src and p-Src(Y416) (**h**) proteins in RT112 and 5637 cells. (**i**) p-Src(Y416)/Src ratio in RT112 and 5637 cells measured after densitometric analysis of Western Blot bands with ImageJ. (**j**) Spheroids formation assay in different supports: tissue culture treated (upper panels), not-treated (middle panels) and cell repellent supports (lower panels). Images were acquired in phase contrast light microscopy. Results are the mean of two (**f**) or three (**b**–**d**,**i**) experimental replicates. Statistical test: *t*-test and linear regression, *** for *p* < 0.001.

RT112 and 5637 (Figure 1a) are bladder cancer cell lines, of the same histological Low Grade (G2). The two cell lines have a similar proliferation rate (Figure 1b), but RT112 cells are significantly larger, as shown by imaging data (average area in square micrometers, Figure 1c) and biochemical determination of the protein content/cell (Figure 1d).

A wound healing assay (Figure 1e,f, Supplementary Video S1) shows that 5637 cells present a significantly higher migration rate than RT112. Consistently, 5637—but not RT112—cells show a significant level of vimentin (Figure 1g), the main protein of intermediate filaments, a canonical marker of epithelial-mesenchymal transition (EMT) reprogramming, associated with the acquisition of migratory and invasive phenotype [45,46]. The increase in the migratory capacity of 5637 is also associated with a reduction in the Src kinase specific activity (here measured as the ratio between Y416-phosphorylated form and the total protein) (Figure 1h,i). This protein plays a key role in the process called “adhesion turnover”, consisting of the continuous formation of cellular matrix at the front pole of the cell and continue old cellular matrix disassembly at the rear [47]. The expression and activity of Src are inversely related to the migratory and metastatic capacity also in other bladder cancer cell lines [48].

A significant difference in the RT112 and 5637 cell lines’ adhesive properties is highlighted by the cells’ different behavior when seeded on polystyrene plates not subjected to the tissue culture treatment, which makes them more hydrophilic, that we refer to as “not treated”. On these plates in serum-free medium, only RT112 cells form spheroids, while the 5637 cell line grows as a monolayer (Figure 1j). On cell repellent plates (in serum-free medium) both cell lines form spheroids. Compared to 5637, RT112 cells form a significantly higher number of spheroids (Supplementary Figure S2a,b), characterized by a significantly larger area (Supplementary Figure S2c).

**Figure 2:**
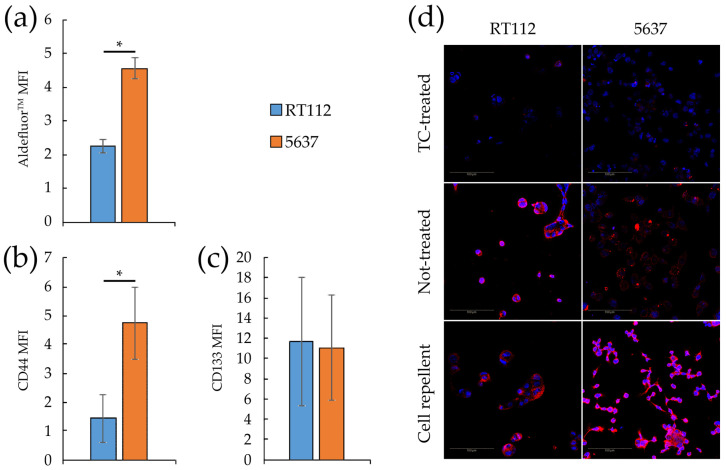
Stemness markers of monolayers and spheroids from RT112 and 5637 cells. (**a**–**c**) Median fluorescence intensity of Aldefluor^TM^ (**a**), CD44 (**b**) and CD133 (**c**) by flow cytometry analysis on RT112 and 5637 cells grown as monolayers. Results are the mean of two (**a**) and three (**b**,**c**) experimental replicates. Statistical test: *t*-test, * for *p* < 0.05. (**d**) Representative images from confocal immunofluorescence (IF) microscopy of RT112 and 5637 cells using SOX2 Antibody (red) and Hoechst 33342 (blue) for nuclei. Cells were grown as monolayer or spheroids on different (Tissue culture-treated, Not-treated or Cell repellent) supports, before being seeded in adherent condition on chamber slides for IF.

Compared to RT112, monolayers of 5637 cells show higher levels of some markers of stemness, and invasiveness, typically linked to tumor malignancy [49,50], namely a higher positivity to the fluorescent dye Aldefluor^TM^ (Figure 2a), a readout of the expression of ALDH1A1 [51,52], and in expression level of the transmembrane glycoprotein CD44 [53,54] (Figure 2b), but not of CD133 [55,56] (Figure 2c). In agreement with previous studies [57], spheroid growth in both lines is associated with increased expression and nuclear localization (activation) of the transcription factor SOX2 (Figure 2d), a member of the SRY-related HMG-box (SOX) family. SOX2 is a known marker of stemness not expressed in healthy urothelial cells and related to the presence of cancer stem cells (CSCs), also in bladder cancer [49].

### 3.2. Glycolytic and Mitochondrial Bioenergetics of RT112 and 5637 Grade 2 Bladder Cancer Cells

Metabolic rewiring is a hallmark of cancer [7,58], allowing cancer cells to meet their energy and biosynthetic needs to support enhanced cell growth and survival under nutrient-poor environmental conditions, not allowing survival or proliferation of normal cells.

The main metabolic routes contributing to the energy homeostasis are glycolysis and oxidative phosphorylation (OXPHOS), which couple the breakdown of nutrients as glucose, amino acids and fatty acids to ATP production. These two pathways also play a pivotal role in redox homeostasis since they contribute to producing the reducing power required for anabolic processes and for counteracting oxidative stress. The Seahorse Extracellular Flux Analyzer (Agilent) measures in real time the ExtraCellular Acidification Rate (ECAR) related to the excretion of lactate that in turn is strictly related to the glycolytic flux, and the Oxygen Consumption Rate (OCR), that is primarily due to mitochondrial respiration, in living cells [59]. The Seahorse XF Glycolysis Stress Test and Mitochondrial Stress Test protocols dissect the glycolytic and respiratory fluxes components into basal, maximal and reserve (spare) glycolytic or respiratory capacity through the consecutive addition of specific drugs, whose optimization is reported in Supplementary Figure S3a–c.

**Figure 3:**
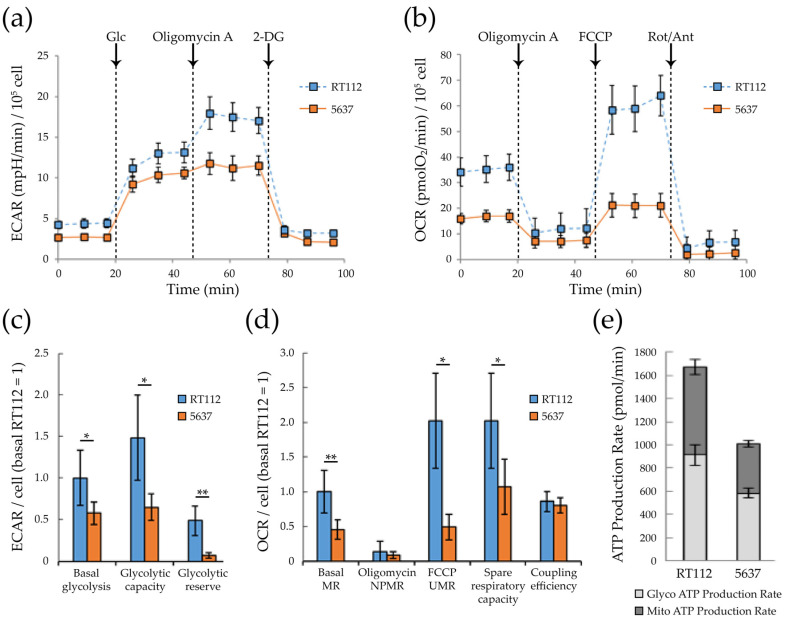
Glycolysis and respiratory bioenergetics of RT112 and 5637 cells. (**a**) Representative ExtraCellular Acidification Rate (ECAR) profile of monolayer cells subjected to XF Glycolysis Stress Test with XF24 Agilent Seahorse under sequential injections of 10 mM glucose, 0.25 µM oligomycin A and 50 mM 2-deoxy-glucose (**b**) Representative Oxygen Consumption Rate (OCR) profile of monolayer cells subjected to XF Mito Stress Test with XF24 Agilent Seahorse under sequential injections of 0.25 µM oligomycin A, 2 µM FCCP and 1 µM Rotenone + 1 µM Antimycin A. ECAR and OCR values measured using XF24 Agilent Seahorse were normalized on protein content measured by Bradford assay. Data were further normalized per cell number, taking into consideration the protein content per cell (Figure 1d) (**c**) Glycolytic bioenergetic parameters measured from Seahorse results: basal glycolysis, glycolytic capacity and glycolytic reserve (**d**). Respiratory bioenergetics parameter measured from Seahorse results: basal mitochondrial respiration (MR), non-phosphorylating mitochondrial respiration (NPMR) and FCCP-uncoupled respiration (UMR), spare respiratory capacity and coupling efficiency. (**e**) ATP production rates due to glycolysis or mitochondrial respiration as measured with XF Real-Time ATP rate assay using XFe96 Agilent Seahorse. Proton Efflux Rate (PER) and OCR values were normalized on cell number measured by counting Hoechst-positive nuclei with Operetta CLS™ and Harmony software. Statistical test: *t*-test, * for *p* < 0.05; ** for *p* < 0.01.

Seahorse analysis demonstrated that, compared to RT112, 5637 cells show a significant reduction in basal and maximal (i.e., oligomycin induced) glycolytic capacity as well as in glycolytic reserve (Figure 3a,c). Consistently, the levels of mRNAs encoding two key glycolytic enzymes are down-regulated in 5637 cells compared RT112: glyceraldehyde 3-phosphate dehydrogenase (GAPDH), responsible of the conversion of glyceraldehyde-3-phosphate to 1,3-biphosphoglycerate with the production of NADH and H+, and lactate dehydrogenase A (LDHA), that catalyzes fermentation of pyruvate to lactate (Supplementary Figure S3d,e). The level of mRNAs encoding the M1 and M2 isoforms of pyruvate kinase M (PKM), that catalyzes the transfer of a phosphoryl group from phosphoenolpyruvate (PEP) to ADP generating ATP, are instead similar in both cell lines (isoform M2, associated with cancer) or higher in 5637 (isoform M1) (Supplementary Figure S3f,g). The physio-pathological consequence of the PKM expression pattern is difficult to predict, since the sugar kinase activity of the enzyme is regulated by a complex interplay between allosteric activators and inhibitors [60,61].

Compared to RT112, 5637 cells show a significantly reduced basal mitochondrial (MR, 2-fold reduction) and FCCP-uncoupled (UMR, 4-fold reduction) respiration (Figure 3b,d). Non-phosphorylating mitochondrial respiration (NPMR) is low and very similar in both cell lines, indicating that the enhanced basal mitochondrial respiration in RT112 is essentially devoted to ATP production. As expected, 5637 cells produce significantly less ATP than RT112 by mitochondrial respiration, and by glycolysis as well (Figure 3e).

**Figure 4:**
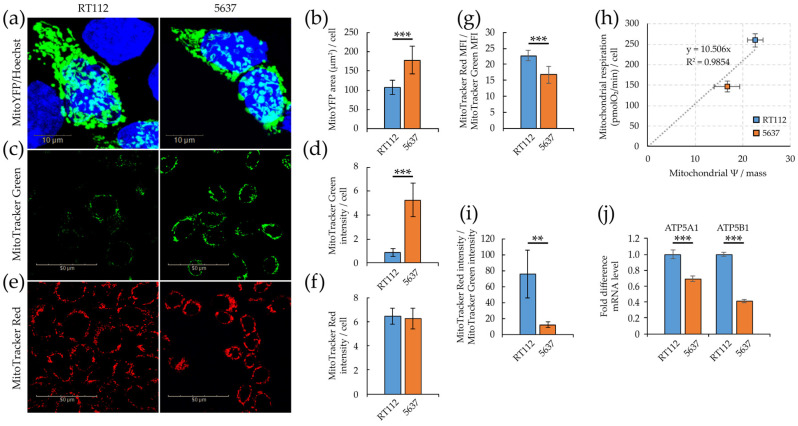
Mitochondrial efficiency of RT112 and 5637 cells. (**a**) Confocal imaging of fixed cells transiently transfected for the expression of the mitochondrial-specific MitoYFP protein (green), stained with Hoechst 33342 dye labeling nuclei (blue). (**b**) Mitochondrial area per cell quantified as the ratio of green (MitoYFP) fluorescent area to blue Hoechst-positive objects (nuclei) in manually selected 30-40 ROIs containing green positive cells. (**c**) Confocal imaging of living cells stained with mitochondrion-selective MitoTracker™ Green FM (green). (**d**) Mitochondrial mass per cell calculated as the ratio of MitoTracker™ green fluorescence intensity to blue Hoechst-positive objects (nuclei). (**e**) Confocal imaging of living cells stained with the potentiometric mitochondrion-selective MitoTracker™ Red CMXRos dye (red). (**f**) Mitochondrial membrane potential per cell calculated as the ratio of MitoTracker™ Red CMXRos fluorescence intensity to blue Hoechst-positive objects (nuclei). (**g**) Mitochondrial membrane potential per mitochondrial mass unit calculated as the ratio of the median fluorescence intensity of MitoTracker Red CMXRos per cell and the median fluorescence intensity of MitoTracker Green per cell by flow cytometry analysis. (**h**) Correlation between basal respiration OCR and mitochondrial membrane potential per mitochondrial mass unit measured by flow cytometry. (**i**) Mitochondrial membrane potential per mitochondrial mass unit calculated by quantitative imaging as the ratio of the Mitotracker Red and Green’s fluorescence intensity per cell. (**j**) mRNA level by qRT-PCR of ATP5A1 and ATP5B1 genes normalized to the house-keeping HPRT-1 gene level. Quantitative imaging analysis was performed with Harmony software (**b**,**d**,**f**,**i**). All results are the mean of at least three experimental replicates. Statistical test: *t*-test, ** for *p* < 0.01; *** for *p* < 0.001.

We further analyzed mitochondrial morphology and function by confocal microscopy and high content analysis performed with Operetta CLS™. We first determined mitochondrial mass using either quantification of a transiently expressed mitochondrial-specific YFP protein (MitoYFP, Figure 4a,b) or staining with MitoTracker Green, a dye which localizes into mitochondria regardless of mitochondrial membrane potential [62] (Figure 4c,d). Quantification of both the MitoYFP- positive area/cell (Supplementary Figure S1a) and of the intensity of the MitoTracker Green signal/cell (Supplementary Figure S1b), suggests that 5637 cells have a higher mitochondrial mass compared to RT112.

We then investigated the mitochondrial membrane potential with MitoTracker Red, a potentiometric fluorescent red dye that accumulates into mitochondria depending on membrane potential, and mitochondrial activity [63]. MitoTracker Red intensity/cell is similar in the two cell lines (Figure 4e,f and Figure S1c). The ratio between the MitoTracker Red and MitoTracker Green intensities determined by imaging (Figure 4i), or the ratio between the median fluorescence intensities determined by FACS (Figure 4g) defines mitochondrial activity per unit of mitochondrial mass (*i.e.*, mitochondrial specific activity). Both measurements indicate that mitochondrial specific activity is higher in RT112 than in 5637 cells and well correlates with enhanced mitochondrial respiration measured by Seahorse flux analysis (basal OCR, Figure 3d), as reported in Figure 4h. In keeping with these data RT112 cells have higher expression level of two ATP synthase subunits, ATP5A1 and ATP5B1 (Figure 4j), and produce more ATP by mitochondrial respiration (Figure 3e).

### 3.3. Redox Homeostasis in RT112 and 5637 Cell Lines

**Figure 5:**
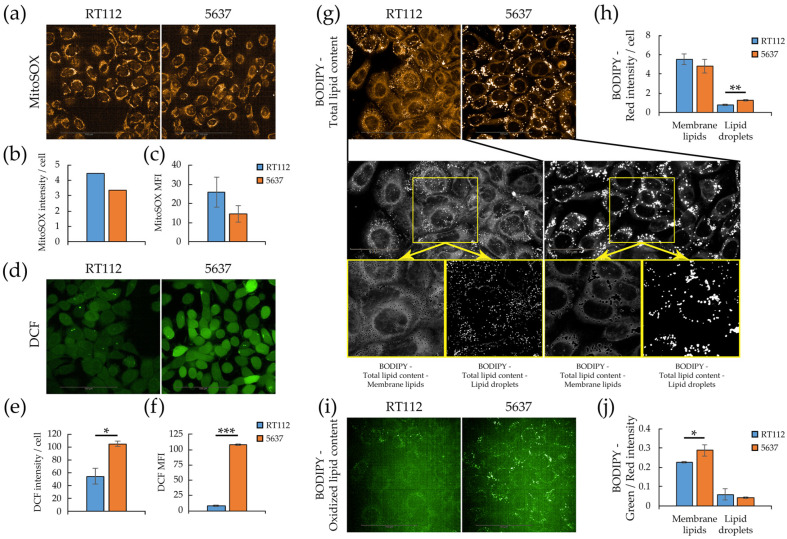
Redox homeostasis and lipid peroxidation in RT112 and 5637 cells. Quantitative imaging was performed with Operetta CLS™ and Harmony software (**a**) Confocal imaging of living cells stained with MitoSOX (orange). (**b**) Mitochondrial ROS level per cell calculated as the ratio of MitoSOX red fluorescence intensity to blue Hoechst-positive objects (nuclei) (**c**) MitoSOX median fluorescence intensity obtained by flow cytometry. (**d**) Confocal imaging of living cells stained with 2′,7′-Dichlorofluorescin diacetate (green). (**e**) Total ROS level per cell calculated as the ratio of DCF green fluorescence intensity to blue Hoechst-positive objects (nuclei). (**f**) Total ROS level per cell obtained from DCF median fluorescence intensity obtained from flow cytometry. (**g**) Confocal imaging of red fluorescence of living cells stained with the Lipid Peroxidation Sensor BODIPY™ 581/591 C11 (orange, total lipid content, upper panels) and grayscale representative image as used for quantitative analysis of total lipid content (middle panels), membrane lipids and lipid droplets (lower panels). (**h**) Lipids per cell quantified as membrane lipids and lipid droplets measured using Harmony software. (**i**) Confocal imaging of green fluorescence of living cells stained with the Lipid Peroxidation Sensor BODIPY™ 581/591 C11 (green, oxidized lipid content). (**j**) Oxidized/total membrane lipids and lipid droplets ratio, calculated as the ratio of green to red fluorescence intensity. All results are the mean of at least three experimental replicates. Statistical test: *t*-test, * for *p* < 0.05; ** for *p* < 0.01; *** for *p* < 0.001.

Mitochondrial respiration is the primary source of cellular ROS and both cell lines under investigation are oxidative. Therefore, we analyzed mitochondrial and cytoplasmatic ROS levels by flow cytometry, and quantitative imaging (Operetta CLS™) on living cells stained with MitoSOX and 2′,7′-dichlorofluorescein diacetate (H2DCFDA), respectively [64]. MitoSOX is a mitochondrion selective dye which is oxidized by superoxide but not by other reactive oxygen species (ROS) and reactive nitrogen species (RNS), while H2DCFDA is a fluorogenic dye that allow measuring hydroxyl, peroxyl and other ROS species within the cell. After passive diffusion into cells, H2DCFDA is deacetylated by cellular esterases to the corresponding dichlorodihydrofluorescein derivative, whose ROS oxidization originates a fluorescent adduct, 2′,7′-dichlorofluorescin (DCF), that remains trapped inside the cells. Quantitative imaging results, confirmed by flow cytometry analysis (Figure 5a–f), showed that although RT112 cells have a higher (30% more) content of mitochondrial superoxide than 5637, in keeping with their higher respiratory rate, they are characterized by a significantly lower total intracellular ROS level.

Since high ROS concentrations lead to free radical mediated chain reactions that indiscriminately target intracellular molecules, including polyunsaturated fatty acids of lipid membranes [65], we analyzed by confocal microscopy (Operetta CLS™) lipid peroxidation using a ratio-fluorescence assay on living cells stained with the oxidative sensitive C11-BODIPY581-591 dye [66]. This probe incorporates readily into cellular membranes and its fluorescence shifts from red to green upon oxidation. Figure 5g,i report confocal images of red and green fluorescence of RT112 and 5637 cells stained with C11-BODIPY581-591. It is possible to distinguish bright fluorescent spots corresponding to lipid droplets, and a diffuse fluorescence corresponding to membrane lipid content. The bottom part of the same panel reports an enlarged, grayscale version of the images to highlight the quantification procedure. Although 5637 and RT112 have a similar total lipid content, quantitative imaging shows that 5463 have a slightly lower membrane lipid content and a lower number of significantly larger and denser lipid droplets on a cellular basis (Figure 5h and Figure S4a,b). In contrast, 5637 show higher oxidation of membrane lipids (diffuse green fluorescence). The two cell lines show no significant difference in lipid droplets’ oxidation (Figure 5j and Figure S4c,d). In keeping with the higher ROS and peroxidized lipids levels, 5637 cells are more sensitive to oxidative stress, namely H_2_O_2_ treatment (Supplementary Figure S4g).

### 3.4. Metabolic Plasticity in RT112 and 5637 Cell Lines

**Figure 6:**
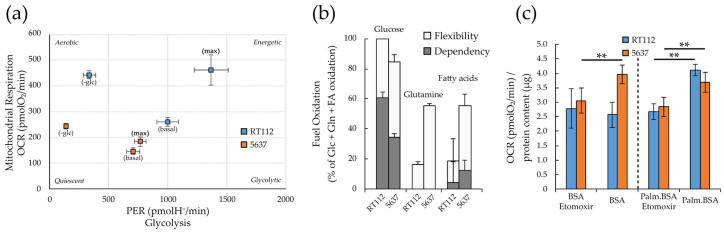
Glycolytic and respiratory capacity and nutrient usage in RT112 and 5637 cells. (**a**) PER and OCR values (attributable to glycolysis and oxidative phosphorylation respectively) in basal condition, maximal capacity (reached with treatment with optimized concentration of oligomycin A for maximal glycolytic capacity or FCCP for maximal respiratory capacity) and under 5 h-glucose deprivation. Seahorse data were normalized on cell number measured after Hoechst 33342 staining as blue-positive objects acquired by Operetta CLS™ and counted using Harmony software. (**b**) The ability to oxidize three different nutrients (glucose, glutamine and fatty acids) reported as dependency and flexibility from XF Mito Fuel Flex Test performed using Agilent Seahorse XFe96 Analyzer. (**c**) OCR values measured in presence/absence of 5 µM Etomoxir from XF Palmitate-BSA FAO Substrate assay using Agilent Seahorse XF24 Analyzer. OCR values were normalized on protein content measured by Bradford assay. All results are the mean of at least three experimental replicates. Statistical test: *t*-test, ** for *p* < 0.01.

As highlighted by the Seahorse experiments described above (Figure 3b,d), RT112 cells are more energetic than 5637, having a more efficient glycolytic and mitochondrial apparatus both for the production of energy in basal conditions, and for responding to eventual energy stress (greater glycolytic reserve, and spare respiratory capacity, or nutrient perturbation). Consistently, when exposed to glucose deprivation (blocking glycolysis) the RT112 cells increase the mitochondrial respiration more effectively compared to 5637. The reduced plasticity of 5637 is appreciable in Figure 6a which reports the two lines’ energy phenotype under basal (in presence or absence of glucose) and maximal conditions (oligomycin-induced glycolysis, and FCCP-uncoupled respiration).

To understand the two cell lines’ ability and flexibility to oxidize different nutrients, we performed the Seahorse XF Mitochondrial Fuel Flex test (Figure 6b). The test showed that both cell lines strongly depend on glucose as a fuel for mitochondrial respiration, make limited use of fatty acids and no use of glutamine at all (dependency, gray bars). Both cell lines are flexible enough to increase allocation of glucose, glutamine and fatty acids for mitochondrial respiration (Flexibility, white bars).

Fatty acid oxidation into mitochondria provides twice as much ATP as carbohydrates on a dry mass basis. Indeed, energy-demanding tissues generally carried out fatty acid β-oxidation (FAO). Furthermore, several cancer cells are dependent on FAO for survival and growth, since it can eliminate potentially toxic lipids, inhibit pro-apoptotic pathways and provide metabolic intermediates for anaplerosis, besides providing ATP and NADPH, counteracting energy and oxidative stress [67].

We extended our studies dealing with energy homeostasis of RT112 and 5637 cells to FAO for these reasons. The Seahorse XF fatty acid β-oxidation (FAO) test assays the cells’ capability to oxidize endogenous fatty acids and/or the long chain fatty acid palmitate, exogenously added in the culture medium. The share of respiration devoted to FAO can be calculated as the difference between the basal respiration and the residual respiration obtained after treatment with 40 µM etomoxir, an irreversible inhibitor of carnitine palmitoyltransferase-1 (CPT1), which is a mitochondrial transporter required for FAO [68]. The FAO assay (Figure 6c) demonstrates that both cell lines can oxidize the exogenous palmitate, while only 5637 cells can also oxidize also the endogenous fatty acids.

### 3.5. Targeting Metabolism in RT112 and 5637 Cell Lines

The different assays reported above (Figure 5; Figure 6) indicate that both cell lines present, albeit with some differences, some kind of metabolic flexibility. Here we tested the pharmacological effect of the inhibition of glycolysis and OXPHOS on cell proliferation, migration and propensity to form spheroids. We used the non-hydrolyzable glucose analog 2-Deoxy-D-glucose (2-DG), to inhibit glycolysis, and metformin to inhibit, possibly indirectly, mitochondrial respiration [69,70,71].

**Figure 7:**
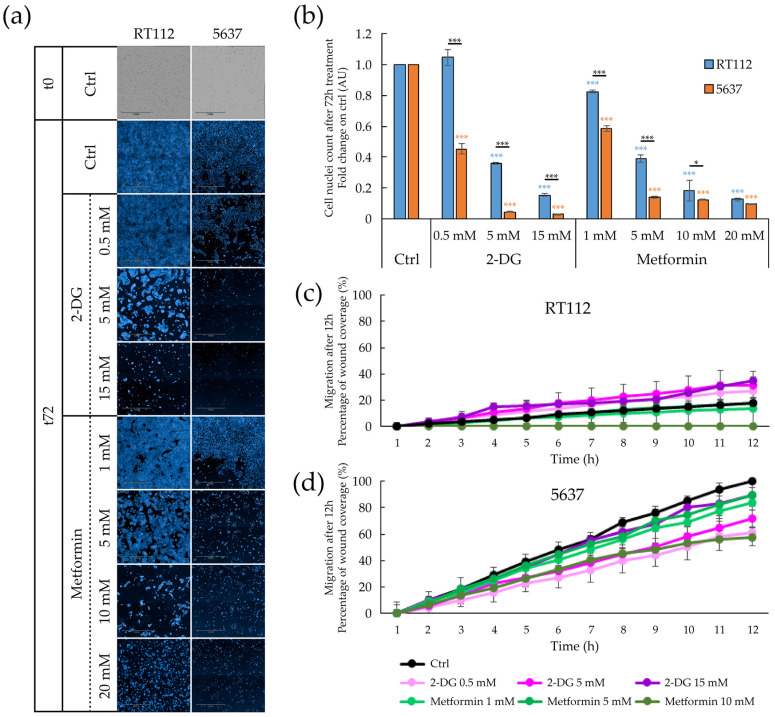
Effect of pharmacological treatment on proliferation and migratory capacity of RT112 and 5637 cells grown as adherent cells. (**a**) Representative images of brightfield (T0) and Hoechst 33342 stained nuclei acquired with Operetta CLS™ of adherent cells after 72 h-treatment with increasing concentration of 2-Deoxy-D-glucose (2-DG) (0, 0.5, 5, 15 mM) and metformin (0, 1, 5, 10, 20 mM). (**b**) Fold change relative to the control condition of nuclei count of cells treated for 72 h grown with 2-Deoxy-D-glucose (2-DG) and metformin treatment. Results are the mean of three experimental replicates. (**c**,**d**) Percentage of wound coverage in wound healing assay after 12 h-treatment condition of increasing concentration of 2-Deoxy-D-glucose (2-DG) (0, 0.5, 5, 15 mM) and metformin (0, 1, 5, 10 mM) of RT112 cells (**c**) and 5637 cells Results are the mean of two experimental replicates, except for 15 mM 2-DG and 10 mM metformin conditions performed in single replicate. Statistical test: *t*-test, * for *p* < 0.05; *** for *p* < 0.001.

Treatment with both 2-DG and metformin inhibits cell proliferation of both bladder cancer cell lines (Figure 7a,b). Compared to RT112, the 5637 cell line’s proliferation was more sensitive to both 2-DG and metformin. This behavior could be due to the more remarkable metabolic plasticity of RT112 cells, which would improve resistance to the inhibition of any of the two energy pathways.

While metformin inhibits in a dose–dependent manner the already limited lateral migration of RT112, 2-DG does not inhibit migration of this cell line (Figure 7c). Indeed, 2-DG seems to paradoxically stimulate cell migration. Whether this is caused by the enhanced metabolic flexibility of RT112 cells or by the short timeframe of this experiment (12 h compared to the 72 h used in the cell proliferation and spheroid formation assays), remains to be seen. Treatments with both metformin and 2DG significantly reduce the migratory capacity of 5637 (Figure 7d).

**Figure 8:**
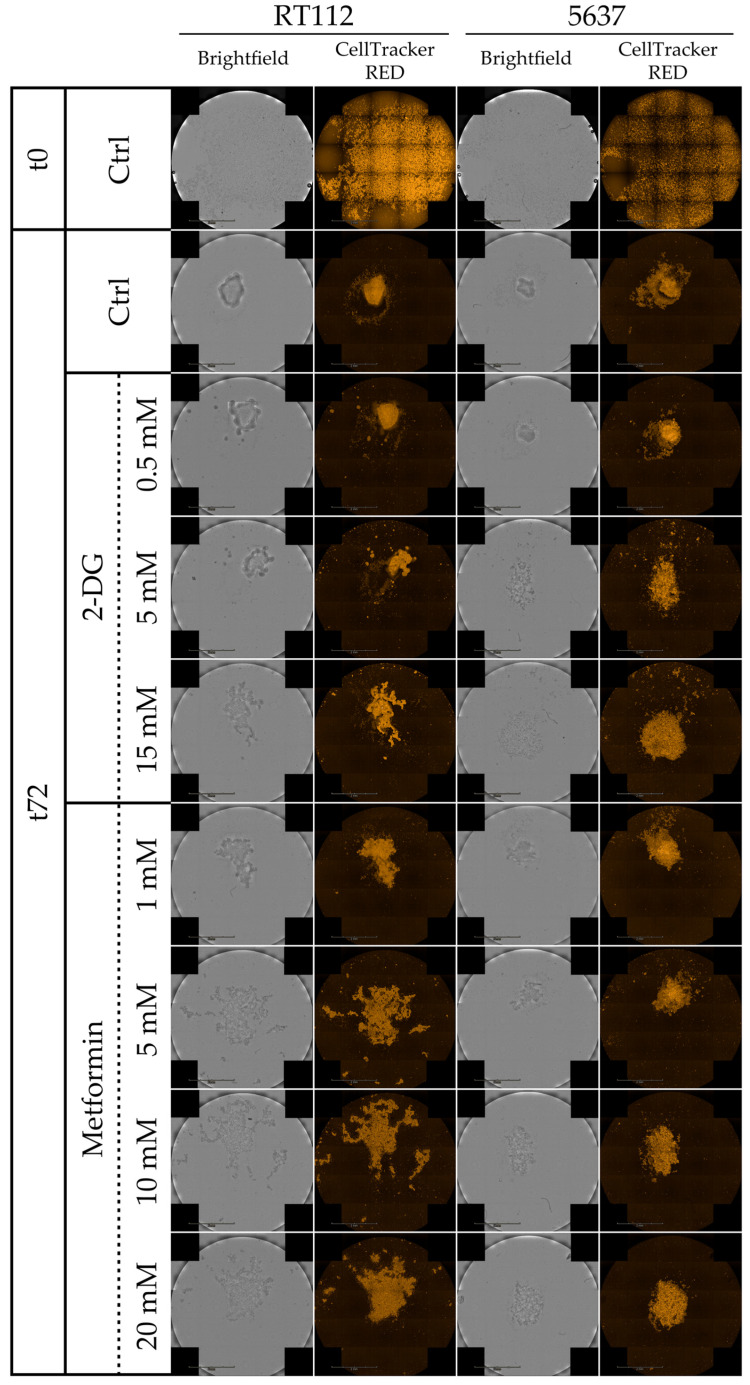
Spheroid formation under pharmacological treatment of RT112 and 5637 cells. Representative images from brightfield and fluorescence microscopy (orange: CellTracker™ Red CMTPX Dye) acquired with Operetta CLS™ at 0 h and 72 h of cells grown in cell repellent 96-well plate under treatment with increasing concentration of 2-Deoxy-D-glucose (2-DG) (0, 0.5, 5, 15 mM) and metformin (0, 1, 5, 10, 20 mM).

Then we tested the effect of 2-DG and metformin on the ability of RT112 and 5637 to form spheroids from single cells in non-adherent conditions. Spheroid formation exploits features of cell progenitors or cancer stem cells (CSC), such as anchorage-independent growth, anoikis resistance and self-renewal [72,73]. Figure 8 and Supplementary Figure S5 show cell tracker-labeled spheroids at 0 and 72 h after seeding and during time course (24-48-72 h) respectively. Untreated spheroids (upper line) have a compact, regular, bright shape and may be surrounded by some cells, especially in the case of the 5637 cell line. The 2-DG treatment has little effect at the lowest tested concentration, while higher concentrations inhibit the development of a properly formed spheroid, originating less bright and more spread aggregates. The effect appears more potent in 5637 cells. A more substantial disruptive effect on the proper formation of spheroids is elicited by treatment with metformin, in keeping with literature data suggesting that spheroid formation is exquisitely dependent on mitochondrial respiration [74,75,76,77]. The aggregates formed after both pharmacological perturbations by RT112 cells appear irregular than those formed by 5637 cells.

## 4. Discussion

Bladder cancer ranks eleventh among the most diagnosed cancer globally. Nearly 90% of these cancers are urothelial carcinomas [78]. Early and accurate diagnostic staging and development of sensitive biomarkers are crucial for identifying the most aggressive cancers and developing appropriate therapeutic interventions. Metabolism is increasing recognized as a driver in the development and maintenance of multifactorial diseases such as cancer [79,80]. Accordingly, detection and characterization of the metabolic footprint of bladder cancers [81,82] could be instrumental for both early diagnosis and therapy monitoring.

Fast cell growth, ability to move and to form 3D structures contribute to defining a cancer cell’s aggressiveness. RT112 and 5637 cells are classified at the same histological low grade, but express molecular markers related to different aggressiveness. The two cell lines present a similar growth rate, but RT112 cells are significantly larger. RT112 form spheroids more efficiently, even on surfaces where 5637 do not form spheroids at all. Even on cell repellent plates, where both cell lines generate spheroids, RT112 form more spheroids with a larger size. In contrast, the 5637 cells migrate much faster (Figure 1 and Video S1). This phenotypic trait is consistent with data that classify RT112 as luminal-like cells enriched in papillary architecture, [43,83,84], and 5637 as basal-like cells enriched for squamous differentiation, considered as models of non-aggressive and aggressive BCs, respectively [44,84,85].

Once characterized the cellular properties associated with spreading and tumor progression of RT112 and 5637, we analyzed their cellular bioenergetics, and nutrient usage in order to identify a potential fragility or dependency to be targeted with a pharmacological treatment.

Seahorse analysis showed that both cell lines can use glycolysis and respiration to produce ATP. However, the RT112 cell line is more energetic than 5637 with a higher basal respiration and glycolytic flux (Figure 6a), essentially due to a potentiated respiratory and glycolytic machinery.

Most cells exploit only a part of their total bioenergetic capacity, operating at a basal level and maintaining a reserve capacity for sudden surges in energy requirements due to stress or increased workload. Under standard growth conditions, 5637 cells use most of their glycolytic and respiratory potential, while RT112 cells have a large, unused glycolytic and respiratory reserve. Both cell lines respond to glucose deprivation by increasing respiration, possibly because glucose starvation unlocks the respiratory machinery. The increase in respiration is less striking in 5637 cells.

Mitochondria are both the source and target of reactive oxygen species (ROS), and damaged mitochondria can release more ROS [86,87]. In detail, about 1–2% O_2_ uptaken by the mitochondria is reduced to superoxide anion radical and ROS as a byproduct of electron transport during oxidative phosphorylation, in particular through the activity of complex I (NADH dehydrogenase ubiquinone-ubiquinol reductase) [88] and complex III (ubiquinol cytochrome c reductase) [89]. Electron transport chain (ETC) generates ROS particularly when is slowed down by high mitochondrial membrane potential (Δψm), or functions in reverse from complex II to complex I, as for ubiquinol excess produced in several mitochondrial metabolic pathways such as fatty acid β-oxidation or oxidation of α-glycerophosphate. ROS generation can also be attributed to the TCA cycle’s specific dehydrogenases due to an increased NADH/NAD+ ratio.

Consistently with their higher respiratory rate, RT112 cells have higher levels of mitochondrial ROS. However, the total level of ROS is higher in 5637 cells. This can be due to a less efficient detoxification system, or poor supply of reducing equivalents required to maintain the antioxidant capacity of the glutathione system. The increased intracellular ROS level in 5637 cells correlates with increased lipid peroxidation. Lipid peroxidation produces highly-reactive aldehyde species, including 4-hydroxy 2-nonenal (4-HNE), considered second messengers of oxidative stress and playing a critical role in cell proliferation and survival, and thereby in cancer progression as reviewed in [90,91,92]. The aldehyde dehydrogenase ALDH3A1 acts as a specific scavenger for these fatty aldehydes [93,94]. RT-PCR analysis (Figure S4h) demonstrates that ALDH3A1 is not expressed in 5637, unlike RT112, indicating that the accumulation of peroxidized lipids could be associated with an ineffective detoxification system. 5637 cells are also more sensitive to H_2_O_2_-induced oxidative stress, suggesting a differential balance between ROS production and detoxification mechanisms in the two bladder cancer cell lines.

Metabolic plasticity is the ability to modulate the use of glycolysis and OXPHOS, to meet the energy and biosynthetic needs depending on the nutritional/environmental conditions, enabling the cancer cells to adapt to various microenvironmental conditions including hypoxia and acidosis. Although most cancer cells, according to the Warburg paradigm, internalize glucose faster and metabolize it via aerobic glycolysis to sustain active proliferation, they remain able to completely oxidize glucose, unless they display significant mitochondrial defects [95]. Indeed both glycolysis and respiration can be simultaneously turned on [9].

In addition to glucose-derived pyruvate, other nutrients provide substrates to the TCA cycle to support mitochondrial metabolism, including lactate and amino acids, i.e., glutamine, and fatty acids. Glucose appears the favorite respiratory substrate for both cell lines, although they can use glutamine and fatty acids when required. Contrary to RT112, the 5637 cell line appears able to use endogenous fatty acids. However, in keeping with the negligible contribution of fatty acids to mitochondria respiration in both cell lines (Figure 6b), in vivo administration of low concentrations of etomoxir [68,96,97]—that guarantee to avoid off-target effects reported at higher dosage [98] and that led to the stoppage of etomoxir clinical trials—does not significantly affect the viability of RT112 and 5637 monolayers (Supplementary Figure S4e,f).

While inhibition of either glycolysis (through treatment with the glucose analog 2-deoxyglucose (2-DG) or respiration (through metformin) results in general down-regulation of cell proliferation, cell migration and spheroid formation in both RT112 and 5637 cells, some difference is worth noting. RT112 cells are less sensitive than 5637 to the inhibitory effect of both drugs on proliferation and cell migration. 2-DG does not inhibit migration in RT112 cells. Migration results with this cell line should be taken cautiously, because of the extremely low migration rate. This overall lower sensitivity to metabolic perturbations is consistent with the more energetic phenotype of the RT112 cell line and its higher ability to cope with metabolic stress, as shown by Seahorse analysis (Figure 3). Metformin appears to inhibit spheroid formation more than 2-DG, in both cell lines. Consistently, recent data indicate that mitochondrial respiration is particularly important in metastatic/circulating cancer cells [99,100], in the development and maintenance of chemoresistance mechanisms [77], and in cancer stem cells [74,101,102], which are particularly enriched in 3D cellular structures, as spheroids.

## 5. Conclusions

In conclusion, our study highlights that two bladder cancer cell lines, established from primary urinary bladder carcinoma of the same histological grade, associated with different aggressiveness and prognoses, present distinct metabolic and invasive properties. Although both cell lines use both glycolysis and respiration to support energy production, RT112 cells present overall higher metabolic plasticity and a higher propensity to form 3D structures while being vastly less efficient in migration. Despite these differences, both cell lines have sizable respiration, and the metformin treatment gives a global down-regulation of the proliferation, migration, and the ability to form spheroids. This suggests that the targeted inhibition of energy metabolism may also be effective in a heterogeneous tumor context. Intriguingly, while RT112 are less sensitive than 5637 to pharmacological perturbation of cell proliferation and migration by 2-DG and metformin, they are more sensitive in the spheroid formation assay. These differences may be partially due to transcriptional rewiring and altered signaling and communication within the forming spheroid architecture. As the molecular and metabolic characterization of these cancers and derived cellular and animal [103] models increase, we will be able to design ever more effective single and combinatorial [104,105] anti-cancer regimens based on the detected metabolic fragilities. This ability appears particularly important for cancers of low histological grade, such as the Grade 2 bladder tumors, representing a critical therapeutic window for adjuvant or post-operative treatments to reduce the tumor mass or avoid the formation of relapses.

## Supplementary Materials

The following are available online at https://www.mdpi.com/2073-4409/9/12/2669/s1: Figure S1: Quantitative imaging by Operetta CLS™ high-content analysis and the Harmony software. Figure S2: RT112 and 5637 cells spheroids formation capacity. Figure S3: Figure S3 Oligomycin and FCCP optimization for the measurement of glycolytic and respiratory bioenergetics, and mRNA levels of selected glycolytic enzymes differentially expressed in RT112 and 5637 cells. Figure S4: Figure S4. Lipid content, lipid peroxidation and redox homeostasis in RT112 and 5637 cells. Figure S5: Spheroid formation capacity of RT112 and 5637 cells under pharmacological treatment; Video S1: RT112 and 5637 cells migration time-lapse.
