# Supplementary material for: Profiling and Targeting of Energy and Redox Metabolism in Grade 2 Bladder Cancer Cells with Different Invasiveness Properties
Source: Cells. 2020 Dec 11;9(12):2669. doi: 10.3390/cells9122669 (PMC7764708; doi:10.3390/cells9122669)
Supplement: Supplementary file 1 [file cells-09-02669-s001.zip › Supplementary_materials.pdf]

cells

MDPI

SUPPLEMENTARY MATERIALS

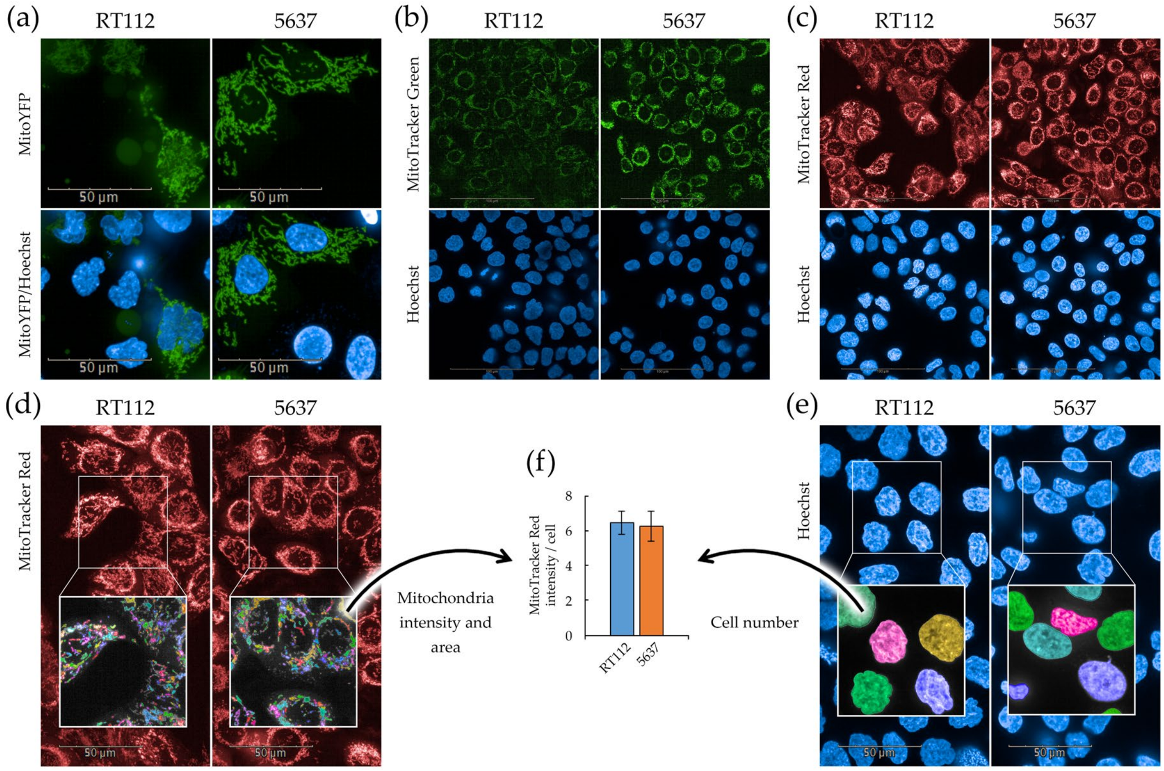

Figure S1. Quantitative imaging by Operetta CLS™ high-content analysis and Harmony software. (**a**) Confocal imaging using Operetta CLS™ of living transiently transfected cells expressing the mitochondrial-specific MitoYFP protein (green) and stained with Hoechst 33342 dye labelling nuclei (blue) showing only MitoYFP on upper panes and merged on lower panes. (**b**) Confocal imaging using Operetta CLS™ of cells stained with MitoTracker™ Green FM dye (green) labelling mitochondria and Hoechst 33342 dye (blue) labelling nuclei. (**c**) Confocal imaging using Operetta CLS™ of cells stained with MitoTracker™ Red CMXRos dye (red) labelling mitochondria and Hoechst 33342 dye (blue) labelling nuclei. (**d**) Confocal imaging using Operetta CLS™ of live cells stained with mitochondrial staining MitoTracker™ Red CMXRos with highlighted mitochondria selection based on MitoTracker™ Red CMXRos fluorescence using Harmony software as operated for imaging analysis to obtain intensity data. (**e**) Hoechst 33342 dye labelling nuclei (blue) and nuclei selection method based on Hoechst 33342 dye fluorescence using Harmony software as operated for imaging analysis to obtain cell count. (**f**) Resulting graph of analysed data representing mitochondrial membrane potential per cell, obtained from MitoTracker™ Red CMXRos fluorescence and Hoechst 33342-positive nuclei count.

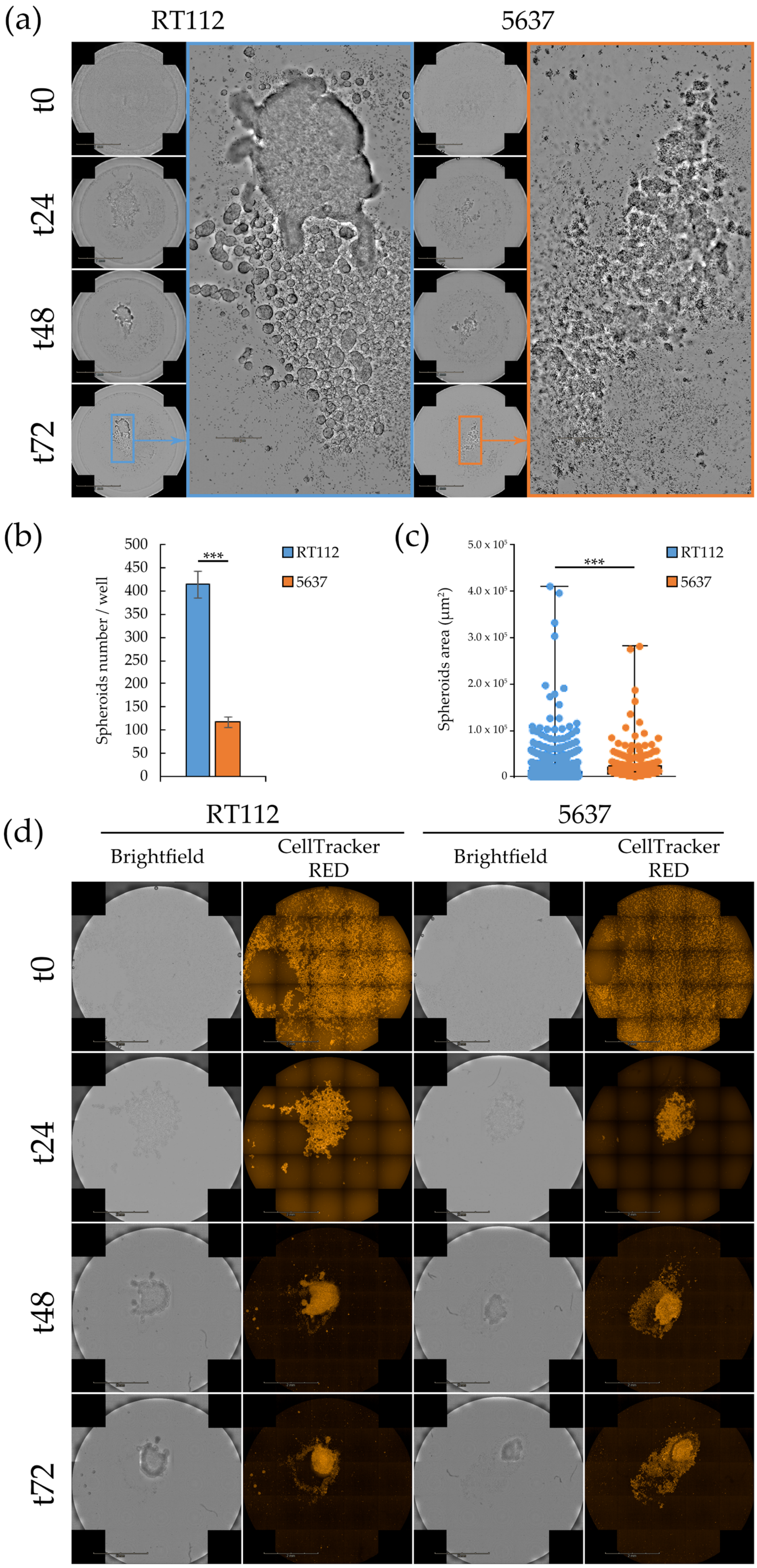
(a)
RT112
5637
t0
t24
t48
t72
(b)
Spheroids number / well
500
450
400
350
300
250
200
150
100
50
0
***
RT112
5637
(c)
Spheroids area (µm²)
5.0 x 10⁵
4.0 x 10⁵
3.0 x 10⁵
2.0 x 10⁵
1.0 x 10⁵
0
***
RT112
5637
(d)
RT112
5637
Brightfield
CellTracker RED
Brightfield
CellTracker RED
t0
t24
t48
t72

Figure S2. RT112 and 5637 cells spheroids formation capacity. (**a**-**c**) Spheroids formation through time (0 hours – 72 hours) in polyHEMA-treated 24-well plate. (**a**) Representative brightfield images acquired using the Operetta CLS™ are shown. (**b**) Number of spheroids per well obtained by manual selection using ImageJ software. (**c**) Whiskers plot of area of spheroids measured on manually selected ROIs using ImageJ software. (**d**) Spheroids formation through time (0 hours – 72 hours) in cell repellent 96-well plate. Representative images of brightfield and fluorescence confocal microscopy (orange: CellTracker™ Red CMTPX Dye) acquired using the Operetta CLS™ are shown. All results are the mean of at least three experimental replicates. Statistical test: t-test, *** for P<.001.

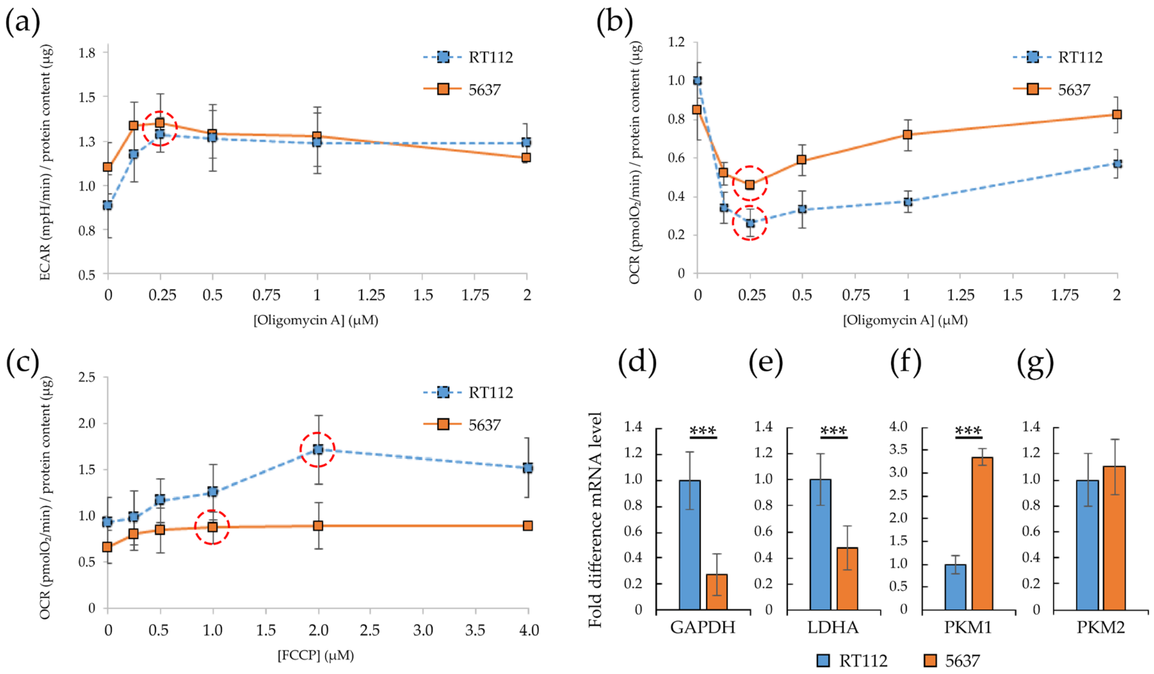

Figure S3. Oligomycin and FCCP optimization for the measurement of glycolytic and respiratory bioenergetics, and mRNA levels of selected glycolytic enzymes differentially expressed in RT112 and 5637 cells. (**a**-**c**) ECAR and OCR values detected by Seahorse XF24 Analyzer normalized on protein content measured by Bradford assay. ECAR (**a**) and OCR (**b**) values measured after injection of increasing concentrations of oligomycin A (0, 0.125, 0.25, 0.5, 1 and 2 µM). OCR (**c**) values measured after injection of increasing concentrations of FCCP (0, 0.25, 0.5, 1, 2 and 4 µM). (**d**-**g**) mRNA level by qRT-PCR of genes GAPDH (**d**), LDHA (**e**), PKM1 (**f**) and PKM2 (**g**) normalized to the house-keeping gene HPRT-1 level. All results are the mean of at least three experimental replicates. Statistical test: t-test, *** for $P<.001$.

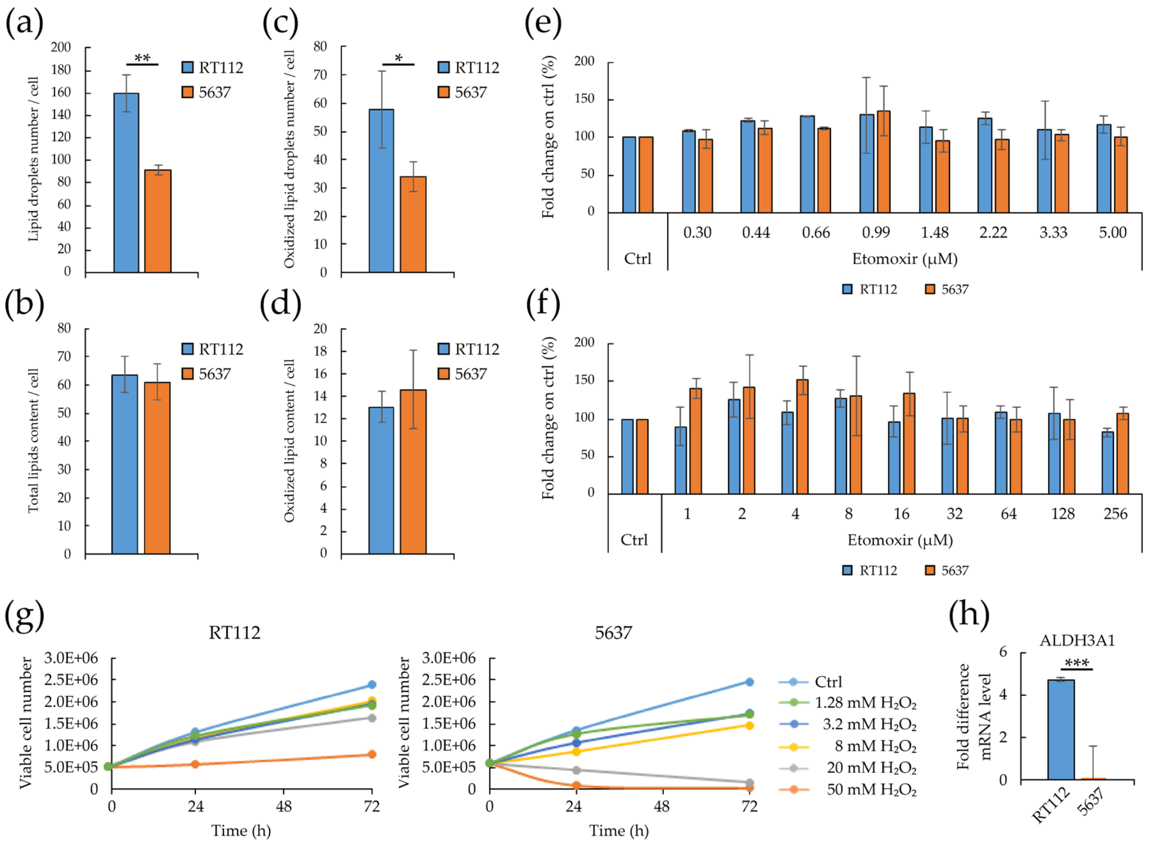

Figure S4. Lipid content, lipid peroxidation and redox homeostasis in RT112 and 5637 cells. (**a**) Number of lipid droplets per cell obtained using Harmony software. (**b**) Total lipids per cell measured using Harmony software. (**c**) Number of oxidized lipid droplets per cell obtained using Harmony software. (**d**) Total oxidized lipids per cell measured using Harmony software. (**e**, **f**) Fold change on control condition of adherent cells treated for 72h with increasing concentration of Etomoxir (e: 0.30, 0.44, 0.66, 0.99, 1.48, 2.22, 3.33, 5 μM; f: 1, 2, 4, 8, 16, 32, 64, 128, 256 μM). (**g**) Growth curves of RT112 and 5637 cells under treatment with increasing concentration of H_2O_2 (0, 1.28, 3.2, 8, 20, 50 mM). Viable cell number at 0, 24 and 72 hours was obtained by the Trypan Blue exclusion method. (**h**) qRT-PCR analysis for ALDH3A1 mRNA levels in RT112 and 5637 cells, normalized to the house-keeping gene HPRT-1 level. All results are the mean of at least three experimental replicates. Statistical test: t-test, * for P<.05; ** for P<.01; *** for P<.001.

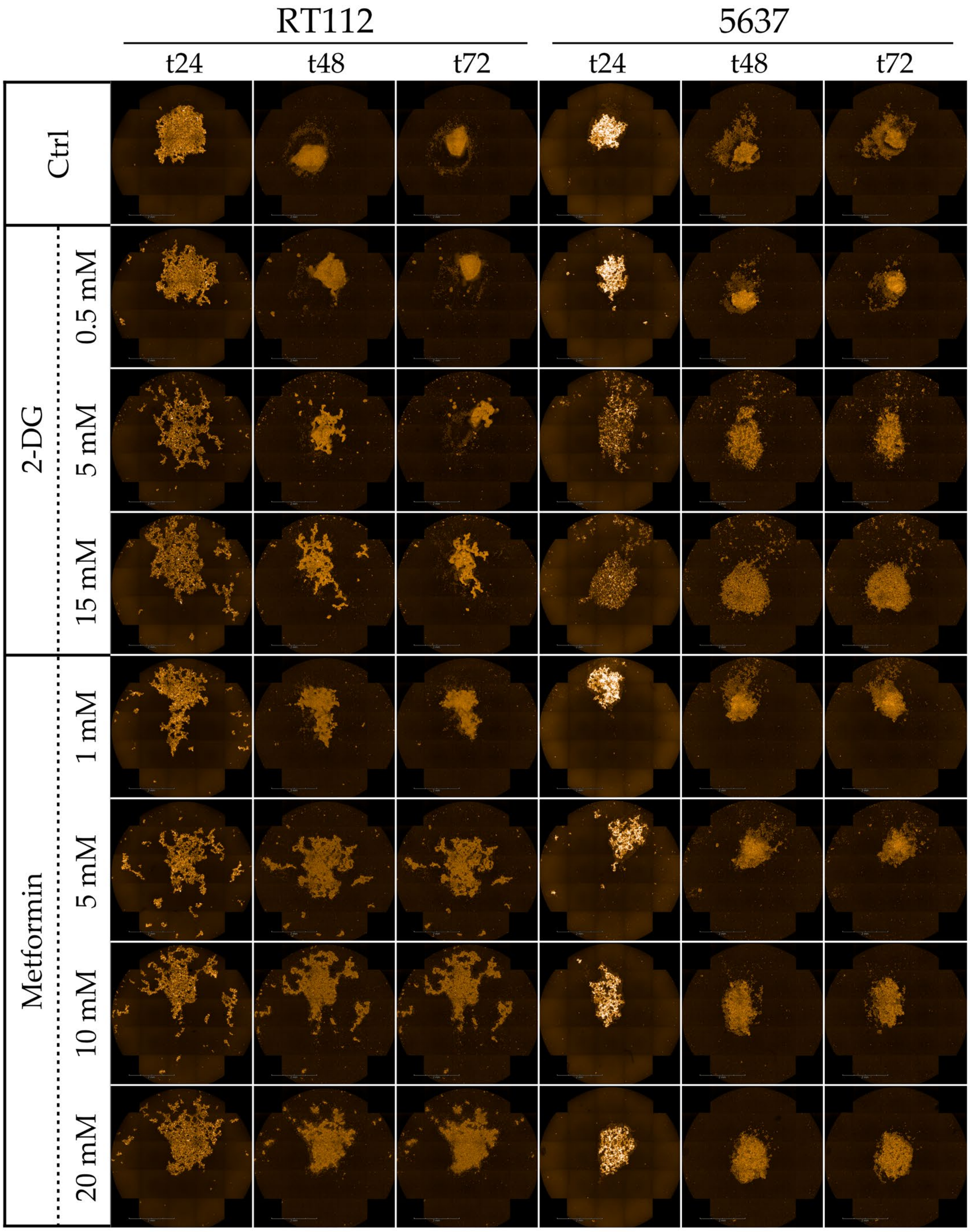

Figure S5. Spheroid formation capacity of RT112 and 5637 cells under pharmacological treatment. Representative images are shown: fluorescence microscopy (orange: CellTracker™ Red CMTPX Dye) at 24, 48 and 72 hours of cells grown in cell repellent 96-well plate treated with increasing concentration of 2-deoxy-D-glucose (2-DG) (0, 0.5, 5, 15 mM) and metformin (0, 1, 5, 10, 20 mM) acquired with Operetta CLS™.

Video S1. RT112 and 5637 cells migration time-lapse. Time-lapse movie showing RT112 (left) and 5637 (right) migration in 12 hour time-course in wound healing assay. Movie frames were obtained with Operetta CLS™ in brightfield and fluorescence microscopy (orange: CellTracker™ Red CMTPX Dye).
